# Disturbed Cardiorespiratory Adaptation in Preeclampsia: Return to Normal Stress Regulation Shortly after Delivery?

**DOI:** 10.3390/ijms20133149

**Published:** 2019-06-27

**Authors:** Helmut K. Lackner, Ilona Papousek, Karin Schmid-Zalaudek, Mila Cervar-Zivkovic, Vassiliki Kolovetsiou-Kreiner, Olivia Nonn, Miha Lucovnik, Isabella Pfniß, Manfred G. Moertl

**Affiliations:** 1 Division of Physiology, Otto Loewi Research Center, Medical University of Graz, 8010 Graz, Austria; 2 Department of Psychology, Biological Psychology Unit, University of Graz, 8010 Graz, Austria; 3 Department of Obstetrics and Gynecology, Medical University of Graz, 8036 Graz, Austria; 4 Division of Cell Biology, Histology and Embryology, Gottfried Schatz Research Center, Medical University of Graz, 8010 Graz, Austria; 5 Department of Perinatology, Division of Obstetrics and Gynecology, University Medical Centre Ljubljana, 1000 Ljubljana, Slovenia; 6 Department of Obstetrics and Gynecology, Clinical Center, 9020 Klagenfurt, Austria

**Keywords:** pregnancy complications, vagal withdrawal, baroreflex sensitivity, blunted cardiac response, cardiovascular adaptations, autonomic nervous system

## Abstract

Women with pregnancies complicated by preeclampsia appear to be at increased risk of metabolic and vascular diseases in later life. Previous research has also indicated disturbed cardiorespiratory adaptation during pregnancy. The aim of this study was to follow up on the physiological stress response in preeclampsia several weeks postpartum. A standardized laboratory test was used to illustrate potential deviations in the physiological stress responding to mildly stressful events of the kind and intensity in which they regularly occur in further everyday life after pregnancy. Fifteen to seventeen weeks postpartum, 35 women previously affected by preeclampsia (19 mild, 16 severe preeclampsia), 38 women after uncomplicated pregnancies, and 51 age-matched healthy controls were exposed to a self-relevant stressor in a standardized stress-reactivity protocol. Reactivity of blood pressure, heart rate, stroke index, and systemic vascular resistance index as well as baroreceptor sensitivity were analyzed. In addition, the mutual adjustment of blood pressure, heart rate, and respiration, partitioned for influences of the sympathetic and the parasympathetic branches of the autonomic nervous system, were quantified by determining their phase synchronization. Findings indicated moderately elevated blood pressure levels in the nonpathological range, reduced stroke volume, and elevated systemic vascular resistance in women previously affected by preeclampsia. Despite these moderate abnormalities, at the time of testing, women with previous preeclampsia did not differ from the other groups in their physiological response patterns to acute stress. Furthermore, no differences between early, preterm, and term preeclampsia or mild and severe preeclampsia were observed at the time of testing. The findings suggest that the overall cardiovascular responses to moderate stressors return to normal in women who experience a pregnancy with preeclampsia a few weeks after delivery, while the operating point of the arterial baroreflex is readjusted to a higher pressure. Yet, their regulation mechanisms may remain different.

## 1. Introduction

Preeclampsia is a pregnancy-specific disorder characterized by sudden onset of hypertension with either proteinuria or end-organ dysfunction, or both, after the 20th week of gestation in a previously normotensive woman, occurring in 3–5% of pregnancies in industrialized countries [1,2].

The pathogenesis of preeclampsia remains poorly understood, even though preeclampsia has been recognized for at least 100 years [3]. In the last 20 years, multiple theories about the ultimate cause of preeclampsia have been developed with little agreement, except for the conclusion that preeclampsia is a multifactorial disease [4,5].

Many approaches have been developed for predicting preeclampsia at an early stage and promising insights have been discovered [5,6,7,8]. Nevertheless, the etiology of preeclampsia remains incompletely understood, although the placenta has been identified as the central organ in the pathogenesis of preeclampsia. Impaired placentation and placental function in early pregnancy remains the leading hypothesis [9,10,11,12], while emerging hypotheses focus on the maternal cardiovascular susceptibility to preeclampsia and pregnancy adaptions [13]. Structural and functional cardiovascular changes were found in women 1 year after preeclamptic pregnancies [14], where the involvement of angiogenic factors such as soluble fms-like tyrosine kinase-1 (sFlt-1) and placental growth factor (PlGF) and placental factors such as placental protein 13 (PP13) and the dysbalance thereof may be used to predict severity and long-term cardiovascular complications of preeclampsia [15,16].

While the identification of etiological factors is without doubt an important task, the management of adverse concomitant effects and consequences of preeclampsia may be even more relevant. Women with pregnancies complicated by preeclampsia appear to be at increased risk of metabolic and cardiovascular diseases in later life, and pregnancy complications and coronary heart disease may have common disease mechanisms [17,18]. As cardiovascular disease (CVD) is a leading cause of death, earlier recognition of those at risk seems vital. Therefore, the diagnosis of preeclampsia, or adverse pregnancy more generally, could be an opportunity for the implementation of primary prevention strategies [17,19,20,21].

Pregnancy is associated with huge cardiovascular and metabolic changes and can be considered as a “stress test” of the somatic and cardiovascular system, suggesting that preeclampsia manifesting in pregnancy is akin to a “failed stress test”. “Failing the stress-test”, that is, absence of the typical autonomically regulated cardiovascular and cardiorespiratory adaptations to pregnancy, may be predictive of cardiovascular disorders in later life, when the system is put under similar strain [21,22].

The autonomic nervous system plays a central role in cardiovascular and cardiorespiratory adaptation to pregnancy-related hemodynamic changes [23,24,25]. Previous research has shown that the increases in peripheral vascular resistance and blood pressure that characterize preeclampsia are mediated, at least in part, by a substantial increase in sympathetic vasoconstrictor activity [26,27]. Autonomic nervous system functioning during pregnancy can be noninvasively assessed by analyzing continuous measures of cardiovascular variables, baroreceptor reflex sensitivity (BRS), and the mutual adjustment of blood pressure, heart rate, and respiration, partitioned for influences of the sympathetic and the parasympathetic branch of the autonomic nervous system [28,29,30,31]. Now, if we can determine abnormalities in blood pressure regulation in affected women at a time at which their preeclampsia is no longer present by definition (i.e., from 12 weeks after delivery onwards) by use of a simple and time-efficient test in the laboratory, this test may be used to evaluate the effectiveness of pharmacological or behavioral interventions for reducing affected women’s cardiovascular risk. Therefore, the aim of this study was to follow up on the regulation of the physiological response to everyday stressful events in preeclampsia several weeks postpartum.

## 2. Results 

### 2.1. Cardiovascular and Hemodynamic Variables

The groups differed in their blood pressure levels and related variables at rest (baseline; mean arterial pressure (MAP), *F*(2,121) = 5.1, *p* < 0.01; systolic blood pressure (SBP), *F*(2,121) = 3.1, *p* < 0.05; diastolic blood pressure (DBP), *F*(2,121) = 4.7, *p* < 0.05), stroke index (*F*(2,121) = 6.1, *p* < 0.01), systemic vascular resistance index (SVRI), *F*(2,121) = 7.3, *p* < 0.01). Bonferroni-corrected post-hoc tests indicated moderately elevated blood pressure levels in the nonpathological range, reduced stroke volume, and elevated systemic vascular resistance in women previously affected by preeclampsia. Group means can be obtained from Table 1.

**Figure 1:**
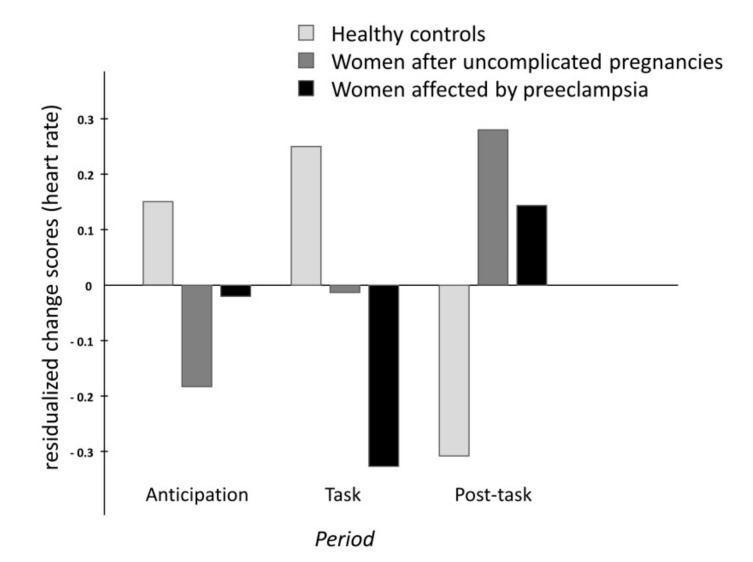
Barplot of the standardized residualized change scores for heart rate (HR).

**Figure 2:**
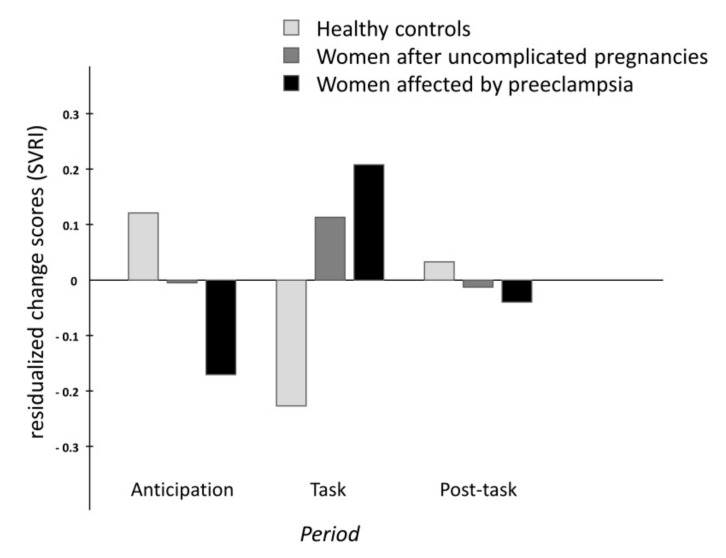
Barplot of the standardized residualized change scores for the systemic vascular resistance index (SVRI).

The analyses of group differences in the time course of cardiovascular changes across the stress manipulation revealed differences between groups in changes of heart rate and systemic vascular resistance (significant interactions period x group). The different time courses are illustrated in Figure 1 (heart rate) and Figure 2 (SVRI). While women without previous pregnancy showed the typical pattern of activation and recovery, women with uncomplicated pregnancies and, even more so, women with former preeclampsia showed blunted responses during the memory task. No significant differences were found for stress-induced changes in blood pressure variables and stroke index. Details of the statistical findings can be found in Table 1.

Women with mild vs. severe preeclampsia did not differ in HR and SVRI levels in resting conditions (baseline; all *p* values >0.504), and no differences were seen among women with early preeclampsia (<34 weeks), preterm preeclampsia (<37 weeks), and women with term preeclampsia (baseline; all *p* values >0.185) Neither did the time courses of changes across the stress manipulation differ between women with mild vs. severe preeclampsia (period x PE-group, all *p* values >0.123; period, all *p* values >0.191), nor were differences seen between early, preterm, and term preeclampsia (period x PE-time, all *p* values >0.221; period, all *p* values >0.144).

### 2.2. Respiration Rate and Baroreflex Sensitivity

The three groups did not differ in respiration rate (*F*(2,121) = 2.3, *p* = 0.107) and BRS (*F*(2,121) = 0.7, *p* = 0.501) in resting conditions (baseline). Changes of respiration rate and baroreflex sensitivity across the stress manipulation did not differ between groups (interactions period x group not significant; for details, see Table 2).

Respiration rate and BRS at rest did not differ between women with mild vs. severe preeclampsia, nor for women with early, preterm, or term preeclampsia (baseline; all *p* values >0.332 and all *p* values >0.665, respectively). Differences between mild and severe preeclampsia in the time courses of changes across the stress manipulation were also nonsignificant (interaction period x PE-group, all *p* values >0.317; main effect period, respiration rate: (*F*(1.5,49.0) = 6.0, *p* < 0.01; BRS: (*F*(2,64) = 4.3, *p* < 0.05)). Differences between early, preterm, and term preeclampsia in the time courses of changes across the stress manipulation were also nonsignificant (interaction period x PE-time, all *p* values >0.330; main effect period, respiration rate: (*F*(1.6,48.6) = 5.5, *p* < 0.05; BRS: (*F*(2,62) = 4.6, *p* < 0.05)).

### 2.3. Adjustment of Blood Pressure, R–R Intervals, and Respiration

#### 2.3.1. Low-Frequency Components

The adjustment of blood pressure and R–R intervals (the interval between consecutive heart beats; RRI) in the low-frequency domain mainly represents the sympathetically modulated mutual interrelation between the two. There were no group differences in resting levels of the synchronization variables γ_SBPxRRI,LF_, γ_DBPxRRI,LF_, and γ_SBPxDBP,LF_ (baseline; *F*(2,121) = 0.8, *p* = 0.461; *F*(2,121) = 0.7, *p* = 0.516; *F*(2,121) = 0.6, *p* = 0.567). Furthermore, the groups did not differ in their stress responses in these variables (Table 3).

Women with mild vs. severe preeclampsia did not differ in these variables (baseline, all *p* values >0.768; period x PE-group, all *p* values >0.271; period, all *p* values >0.446). No significant results were seen for women with early, preterm, and term preeclampsia (baseline, all *p* values >0.460; period x PE-time, all *p* values >0.098; period, all *p* values >0.591) either.

#### 2.3.2. High-Frequency Components

The adjustment of blood pressure, R–R intervals, and respiration in the high-frequency domain represents the parasympathetically modulated mutual interrelations. In resting conditions, the groups did not differ in the synchronization variables γ_SBPxRRI,HF_, γ_DBPxRRI,HF_, γ_RESPxRRI,HF_, γ_RESPxSBP,HF_, and γ_RESPxDBP,LF_ (baseline, *F*(2,121) = 0.9, *p* = 0.43; *F*(2,121) = 0.5, *p* = 0.624; *F*(2,121) = 0.1, *p* = 0.917, *F*(2,121) = 0.2, *p* = 0.795; *F*(2,121) = 0.7, *p* = 0.523). No significant differences were observed in the changes of these variables during the stress manipulation (Table 4). However, some statistical trends emerged for the adjustment of respiration and blood pressure, which seemed to be attributed to the women affected by preeclampsia. 

Women with mild vs. severe preeclampsia did not differ in these variables (baseline, all *p* values >0.199; period x PE-group, all *p* values >0.712 except for adjustment of respiration and R–R intervals, *p* = 0.084, and for respiration and systolic blood pressure, *p* = 0.100; period, all *p* values >0.199). Women with early, preterm, and term preeclampsia did not differ in these variables (baseline, all *p* values >0.649; period x PE-time, all *p* values > 0.162; period, all *p* values > 0.571).

### 2.4. Supplementary Analyses

No differences in chronic stress experience were seen between women with a history of preeclampsia (PE), women with uncomplicated pregnancies (UP), and age-matched women without gestation (CO) (*F*(2,121) = 0.7, *p* = 0.507). The rating of how difficult and how stressful the participants had perceived the task to be showed no differences between the groups (difficult, *F*(2,211) = 0.1, *p* = 0.932; stressful, *F*(2,211) = 0.2, *p* = 0.802). Furthermore, no differences in depressive symptoms were seen (*F*(2,121) = 0.2, *p* = 0.784).

## 3. Discussion

The results of the present study showed that the short-term blood pressure regulation in women previously affected by preeclampsia returns to normal several weeks postpartum. However, heart rate and systemic vascular resistance responses in women previously affected by preeclampsia indicated impaired ability to flexibly respond to moderate stress; this indicates that their overarching regulation mechanisms may be altered after all.

In the healthy organism, physiological mechanisms of blood pressure regulation maintain the arterial blood pressure at a largely constant level even in stressful conditions, ensuring adequate tissue perfusion throughout. However, the intense hemodynamic modifications during pregnancy result in a decrease of baroreceptor sensitivity; this is even more pronounced in preeclampsia, and it jeopardizes the proper adjustment of the physiological factors regulating the arterial blood pressure [29,32]. Impaired baroreceptor sensitivity can still be observed after pregnancy, depending on the time since delivery. Walther et al. reported reduced baroreflex sensitivity four days after delivery and concluded that the maternal cardiovascular system is still affected by pregnancy at that time [33]. With greater distance since delivery, BRS returns to levels recorded at the beginning of pregnancy [28,34]. The present findings indicate that this similarly applies to women who have had preeclampsia. Fifteen to seventeen weeks after delivery, the BRS of women with pregnancies complicated by preeclampsia did not differ from that of mothers with uncomplicated pregnancies, and neither did it differ from the BRS of women who did not give birth. Thus, persistently impaired BRS does not seem to explain the increased cardiovascular risk in later life of women who have had preeclampsia.

The assessment of BRS provides some indication of the regulatory activity of the autonomic nervous system in stressful conditions, but for more specific information, more profound analysis of the regulating factors is required. Important additional information is provided by more fine-grained analysis of the variations of the state of the complex regulatory system over time. Phase synchronization indexes, which indicate mutual adjustments of blood pressure, R–R intervals, and respiration across time, supply this information [35,36]. However, in the present study, mutual adjustment of the mainly sympathetically modulated low-frequency variations of heart rate and systolic blood pressure did not differ between groups, thus basically confirming the findings obtained from the BRS.

Nevertheless, the conclusion that blood pressure regulation has entirely returned to normal several weeks postpartum is still premature. From the mathematical approach for the estimation of BRS alone, it follows that baroreceptor sensitivity decreases with increasing levels of heart rate. While some heart rate acceleration during demanding conditions is adaptive, not all individuals may show this adaptation to the same extent. In other words, it is vital to also consider the contributing factors when interpreting the presence or absence of differences in baroreceptor sensitivity between groups (i.e., heart rate, stroke volume, and systemic vascular resistance). 

To maintain a constant arterial blood pressure—the primary regulated variable in stressful situations—increases of heart rate (or stroke volume) must be compensated by decreased vascular resistance in line with the fundamental Darcy’s law (or Ohm’s law) of hemodynamics. In preeclamptic women, factors such as PP13, responsible for vasodilation and decreased vascular resistance, are reduced early in gestation [37]. Their dysregulation may thus not only be involved in endothelial dysfunction during pregnancy, but markers such as PlGF and sFlt-1 may also function as predictors for long-term cardiovascular health [38]. Later in pregnancy, PP13 is massively increased and returns to basal levels within two weeks in healthy women, while it takes more than eight weeks until it returns to normal levels in women who experienced preeclampsia [39]. Since the effects of PP13 last even after its disappearance, PP13 might still influence the maternal vascular system months after pregnancy [37]. Maybe only then do the true effects of preeclampsia on the maternal system become obvious.

In the present study, heart rate markedly increased and vascular resistance decreased accordingly in response to the stress manipulation only in women without previous pregnancy. By contrast, women who were pregnant, and in particular women with former preeclampsia, hardly showed any heart rate responses at all. Hence, in women recovering from preeclampsia, there was no necessity for short-term blood pressure regulation via activation of baroreceptors and subsequent adaptation of vascular resistance. From this, it follows that the similar outcomes in healthy women and women with preeclampsia in terms of baroreceptor sensitivity and maintenance of largely constant arterial blood pressure levels during brief periods of stress do not justify the conclusion that blood pressure regulation is unimpaired after preeclampsia. Instead, it appears to be that blunted cardiac responses in affected women did not make blood pressure regulation necessary to the same extent as in healthy women.

In women without previous pregnancy, the flexible cardiac response to the active performance task, which provides adaptive energy mobilization and oxygen supply, indicates proper functioning of their autonomic nervous system-mediated cardiovascular regulation [40]. In contrast, the blunted heart rate response in women previously affected by preeclampsia may be related to their generally elevated levels of systemic vascular resistance, and the more rigid vasoadaptation associated with this. This idea is fueled by the elevated systemic vascular resistance levels in women with a history of preeclampsia observed at baseline, which is in line with previous research [41].

Various proinflammatory factors, such as TNF-alpha, IL-10, IL-6, leptin, and CX3CL1, are elevated in preeclamptic women [42,43,44] and are also known to mediate cardiovascular remodeling outside of pregnancy, in crosstalk with reactive oxygen species, angiotensin II, and other proinflammatory cytokines [45,46,47,48,49]. In pregnancy, vascular remodeling may also be induced by an elevated maternal inflammatory profile. TNF-alpha induces collagen I deposition in the maternal vasculature, and MMP1 and -7 activity induce extracellular matrix degradation [50], while CYP2J2, elevated in preeclampsia, may also be involved in uteroplacental and vascular remodeling [51].

Thus, the blunted cardiac responses in women with former preeclampsia may arise from an attempt by the organism to protect itself against undue elevations of arterial blood pressure, which would occur as a result of the failure to lower the systemic vascular resistance synchronously with the rising heart rate (and/or stroke volume). With more severe stress as well as in later life when, due to normal aging alone, vasoadaptation becomes even more rigid, this compensatory mechanism may not suffice to prevent harmful elevations of blood pressure. One factor that may account for basally elevated levels of systemic vascular resistance in preeclampsia is the influence of endothelium-derived vasoconstrictors [20], which are linked to endothelial dysfunction and may be key components in the etiology of preeclampsia [52,53]. Impaired endothelial nitric oxide synthase (eNOS) function and decreased NO action, and on the other hand, overrepresentation of inflammatory and antiangiogenic factors, contribute to a preeclamptic phenotype. Restoring this balance is currently in focus for new therapeutic approaches such as capturing or silencing sFLt-1, and although still inconclusive, evidence also shows that the NO donor and vasodilator pentaerythritol tetranitrate (PETN) reduces the incidence of preeclampsia in a high-risk study population [54,55,56]. The vasoconstrictor angiotensin II mediates stimulation of factors such as human placental lactogen and sFlt-1, as well as transcription of inflammatory cytokines through angiotensin II type 1 receptor activation (AT1R) [57,58]. Increased agonistic autoantibodies against AT1R (AT1-AA) in preeclampsia [59,60] also contribute to AT1R activation, whereas AT1-AA blockade was shown to reduce preeclamptic symptoms in rats [61]. Other endothelium-derived factors promote monocyte adhesion and migration by monocyte chemotactic protein-1 (MCP-1) and intercellular adhesion molecule-1 (ICAM-1) expression, for example, contributing to vascular inflammation [62]. Hypertension-induced shear stress can induce endothelium-derived vasoconstrictors such as endothelin-1 and angiotensin II [63,64]. These do not only regulate the vascular tone, but also vascular smooth muscle cell (VSMC) growth and migration into the intima [65,66], where they proliferate in response to cytokines and growth factors such as platelet-derived growth factor (PDGF) [67]. Angiotensin II, endothelin-1, and other cytokines lead to VSMC dedifferentiation to a migratory proliferative type that is fundamental to vascular pathogenesis and remodeling [65,68], which eventually leads to collagen and elastin deposition by VSMCs into the intima and increased vascular rigidity [69]. 

When looking more closely at the contribution of the autonomic nervous system, it is important to note that scientists primarily focus on sympathetic nervous system control, although parasympathetic regulation appears to play an important role in pregnancy [70]. Moreover, as heart rate levels remain below the intrinsic heart rate, moderate active performance stressors such as the one used in the present study primarily trigger parasympathetically mediated cardiorespiratory adaptation, i.e., fast vagal withdrawal [29,35]. Another plausible explanation for the blunted heart rate responses to stress in women with former preeclampsia is impaired adaptation to changing demands through vagal withdrawal, brought about by subordinate functioning of the parasympathetic branch of the autonomic nervous system. This notion is corroborated by the present finding that the mutual adjustments of respiration and heart rate as well as the mutual adjustments of respiration and blood pressure during the stress manipulation tended to be lower in women with previous preeclampsia compared to healthy women. In line with that, previous research has shown that sympathetically mediated regulation prevails during pregnancy, and it has been suggested that impaired parasympathetic regulation along with sympathetic overactivity may contribute to the pathophysiology of preeclampsia [23,26,71]. 

Taken together, arterial blood pressure was maintained at a largely constant level throughout the stress manipulation in healthy women as well as in women with history of preeclampsia; however, different reasons seemed to have accounted for this outcome.

No differences were observed between women with severe compared to mild preeclampsia. However, while severity of preeclampsia was defined in the current study by recommended criteria, these criteria used to differentiate severe from mild preeclampsia are currently a subject of debate [12]. A further limitation may be that on average, women with preeclampsia had greater body mass, which is an important determinant of cardiovascular function. However, it should be noted that stroke volume and systemic vascular resistance were calculated relative to body surface area, thus mitigating the potential confoundment, and results remained unchanged if the variables were adjusted for body mass index (BMI). Nevertheless, possible effects of body mass cannot be completely ruled out. On the other hand, body mass did not differ between women with preeclampsia and women with uncomplicated pregnancies. The groups did not differ in parity either, which may also impact the development of blood pressure in later life [72]. In addition, the fact that we have no information about the women’s stress responses in the time before they became pregnant and the relatively small sample size should also be considered as important limitations of this study. As the risk factors for preeclampsia are the same as those predisposing to cardiovascular disease and might have the same effect on stress responses, problems may already precede pregnancy and then persist after delivery. The latter to some extent limits the interpretation of the analysis, especially within the women with preeclampsia. Due to the relatively small sample size, some analyses are at the lower limit with regard to the statistical power, in particular those analyzing differences between subtypes of preeclampsia. We hope that reporting these results nevertheless may encourage other researchers to use similar methods in larger cohorts of women with former preeclampsia, while separating women according to the subtypes of preeclampsia. Furthermore, there are a variety of excellent methods for the description of important metabolic and vascular changes during pregnancy and beyond which the present study did not include. Lack of such variables without doubt limits the overall explanatory power of the study. However, the point of the present study was to introduce a different approach that represents a novel addition to these metabolic and vascular variables. The approach of the present study provides new and additional information that is not easily gained from metabolic and vascular variables, particularly with regard to the functionality of the neuro-regulation of cardiovascular adaptation on a short-term basis. 

Recent longitudinal assessment of preeclampsia has suggested masked hypertension at 12 weeks postpartum [73]. In line with this observation, the present findings indicated moderately elevated blood pressure levels in women who were previously affected by preeclampsia. This suggests that the operating point of the arterial baroreflex may remain readjusted to a (slightly) higher pressure several weeks after delivery, although all blood pressure levels in the present study were in the nonpathological range. More importantly, the present study highlights that the identification of altered regulation mechanisms and impaired functioning of specific elements of the cardiovascular regulatory circuit in women with history of preeclampsia may be of greater scientific and finally clinical prognostic value than the monitoring of blood pressure levels and baroreceptor functioning alone. As preeclampsia has become a well-recognized risk factor for life-threatening cardiovascular complications in later life, several attempts have been made to include obstetric history in routine screening of cardiovascular risk. In the future, standardized stress response testing may significantly add to the identification of risk. Given the relatively novel stress parameters evaluated here, it appears that according to such novel lab testing to evaluate increased risk of developing cardiovascular complications (with or without relevant symptoms for metabolic syndrome), the risk is not clearly established early after delivery in women who experience preeclampsia during their pregnancy. Further studies are warranted to verify these findings and also to identify when such stress measures tested here become indicative of future cardiovascular complications for women who experienced preeclampsia. Finally, to date, very little guidance exists on the use of tailored prevention strategies in women with history of preeclampsia.

## 4. Materials and Methods 

### 4.1. Participants

The study sample included 35 women with a history of preeclampsia (PE), 38 women with uncomplicated pregnancies (UP), and 51 age-matched women without gestation during the last three years (CO). Women with preceding pregnancy (PE and UP groups) were asked to participate in the study 13–15 weeks postpartum and were tested 15–17 weeks after delivery.

For twenty-one women with an uncomplicated pregnancy (UP), it was their first child (while in PE, *n* = 22); for 14, it was their second child (in PE, *n* = 10); for two, it was their third child (in PE, *n* = 3); and for one woman, it was her fourth child. Parity did not differ between women with uncomplicated pregnancies and women with a history of preeclampsia (χ^2^ = 1.9, *p* = 0.59). Of the 35 PE pregnancies, 19 were diagnosed with mild PE and 16 had symptoms of severe PE. In 6 PE pregnancies, gestation was terminated before 34 weeks (early PE), between weeks 34 and 37 (preterm preeclampsia) in 14 PE pregnancies, and 15 children were delivered after 37 weeks (term PE). One woman with a history of preeclampsia, two women with uncomplicated pregnancies, and 15 women without gestation were smokers. All women were of Caucasian ethnicity. Characteristics of the study sample are presented in Table 5.

Preeclampsia was confirmed using the recommendations of the American College of Obstetricians and Gynecologists Task Force on Hypertension in Pregnancy (2013) [74]. Inclusion criteria were: Systolic blood pressure ≥140 mmHg and/or diastolic blood pressure ≥90 mmHg, presenting at ≥20 weeks gestation and returning to normotensive values within 12 weeks postpartum, blood pressure measured twice and at least 4 h apart. Proteinuria: either protein ≥300 mg per 24 h urine collection, or protein/creatinine ratio ≥0.3, or protein ≥30 mg/dL or 1+ on urine dipstick. Severe preeclampsia was confirmed in the same way as preeclampsia, as defined above, except one of the following had to be present and proteinuria was not required: (1) systolic blood pressure ≥160 mmHg, measured twice at least 15 min apart; (2) diastolic blood pressure ≥110 mmHg, measured twice at least 15 min apart; (3) thrombocytopenia: platelet count <100,000/microliter; (4) impaired liver function: AST or ALT ≥70 units/L or twice the normal concentration; (5) renal insufficiency: serum creatinine ≥1.1 mg/dL or doubled from baseline values; (6) pulmonary edema; or (7) symptoms indicating possible cerebral or neurologic involvement: headache or visual changes (e.g., flashing, blurring, visual loss, blindness). Participants with uncomplicated pregnancies had singleton pregnancies with term delivery. Exclusion criteria in both groups with preceding pregnancy and the age-matched women without gestation were: diabetes mellitus, renal disease, chronic hypertension, antiphospholipid antibody syndrome, kidney transplant, hypothyroidism, thyroid antibodies, pre-existing cardiovascular problems, and seizures. Women with multiple gestations or substance abuse (alcohol, tobacco, illegal drugs) were also excluded.

Written informed consent was provided for all participants included in the study. The study was approved by the authorized ethics committee, Medical University Graz, Austria (No. 27-515 ex 14/15, *Pregnancy complications: challenge and/or chance for further cardiovascular risk in later life?*, date of approval: 14 September 2015) and the Ethics committee Carinthia, Austria (No. A16/15, *Pregnancy complications: challenge and/or chance for further cardiovascular risk in later life?*, date of approval: 10 September 2015)

### 4.2. Experimental Procedure

The experiment started with an approximately 30 min period in which the participating women could adapt and settle down. In this period, general questions were asked, electrodes were attached, and the electrophysiological signals were checked. The fully automated study protocol including highly synchronously transmitted physiological signals started with the perceived stress questionnaire, to measure chronic stress experience, and the depression scale (CES-D), as well as some other questionnaires that are not relevant to this paper [75,76]. After a 5 min resting period in which the participants were asked to remain seated, not to speak, and to relax, the memory task was explained using a prerecorded auditory instruction backed by corresponding information on a computer screen. To increase the self-relevance of the task and hence its stressful character, participants were told that their test performance would be evaluated by colleagues from the psychiatry department who would determine whether their mnemonic abilities corresponded to their age or if they indicated premature aging of the brain. The task was to recall as many words as they could from a list of words taken from a standardized memory test [77]. After the participants had confirmed that they had understood the instruction, the memory task was provided in a fully automated manner. Following completion of the memory task, there was another 5 min relaxation period, and participants subsequently rated how difficult and how stressful they had perceived the task to be, on two 17-point rating scales (ranging from “not difficult at all” to “extremely difficult” and from “not stressful at all” to “extremely stressful” [78,79,80]. Participants remained seated during the entire study protocol.

### 4.3. Data Acquisition and Preprocessing

Continuous hemodynamic monitoring of blood pressure (BP), heart rate (HR), and thoracic impedance was carried out with the Task Force Monitor^®^ (TFM^®^; CNSystems, Graz, Austria) throughout the entire test procedure. HR (3-lead electrocardiography; sampling rate = 1 kHz) and thoracic impedance (sampling rate = 50 Hz) were recorded using specific CNSystems electrodes placed at the neck and the thoracic region, the latter specifically at the midclavicular line at the xiphoid process level. Continuous BP (sampling rate = 100 Hz) was derived from the finger using a refined version of the vascular unloading technique and corrected to absolute values with oscillometric BP measurement on the contralateral upper arm [81]. For analyzing the cardiovascular regulation, software-tools developed in MATLAB^®^ (MathWorks, Natick, MA, USA) were used [29,82].

To obtain R–R intervals (RRI) and blood pressure time series with equidistant time steps, the beat-to-beat values were resampled at 4 Hz, using piecewise cubic spline interpolation after semiautomatic artifact correction. Single artifacts were replaced by interpolation [82]. Furthermore, the respiratory signal was derived from the thoracic impedance and downsampled to 4 Hz to obtain corresponding sampling times as RRI and BP.

The sequence technique was used for the assessment of baroreceptor reflex sensitivity (BRS) [83]. This technique is based on identifying consecutive cardiac beats in which an increase in systolic blood pressure is accompanied by an increase in RRI, or in which a decrease in systolic blood pressure is accompanied by a decrease in RRI. The regression line between the systolic blood pressure and RRI produces an estimate of BRS. We defined an equivalent change in heart rate and systolic blood pressure for at least three consecutive cardiac cycles as a regulatory event if the following criteria were fulfilled: RRI variations >4 ms and systolic blood pressure changes >1 mmHg [31].

### 4.4. Analysis Procedure Using Phase Synchronization

The analysis of synchronization, e.g., of R–R intervals and systolic blood pressure, is based upon the weak coupling of two different systems, which can be analyzed using the concept of analytic signals [29,35,84]. For this purpose, a phase (but not its amplitude) needs to be defined for a time series that contains oscillations in a narrow frequency band. That is, the adjustments of the rhythms of the R–R intervals, blood pressure, and respiration were partitioned for the sympathetic and the parasympathetic branches of the autonomic nervous system [29]. To permit a clear physical interpretation, we used the Hilbert transform to compute the so-called discrete-time analytic signal *X*_D_, with *X*_D_ = *X*_R_ + *i·X*_I_ such that *X*_I_ is the Hilbert transform of the real vector *X*_R_, from the band-pass filtered time series. Subsequently, the phase of the resulting signals *X*_D1_(*t*_i_) and *X*_D2_(*t*_i_) at every time point t_i_ and the difference between these two given phase vectors for the interpolated bivariate data series, e.g., between heart rate and respiration, was calculated.

The time series are defined as synchronized if the phase difference Ψ(*t*_i_) is constant over time. In case of synchronization, the distribution of the phase difference, quantified by the synchronization index γ = {cos(Ψ(*t*_i_))}^2^ + {sin(Ψ(*t*_i_))}^2^, shows a definite maximum. Theoretically, if the synchronization index γ = 1, then both time series are completely synchronized in a statistical sense, while in the case of γ = 0, both time series are completely desynchronized. Thus, the analysis of phase synchronization provides a quantitative indicator of the coordinated behavior of pairs of systems (i.e., in this case, R–R intervals and systolic blood pressure, partitioned for the sympathetic and the parasympathetic branches of the autonomic nervous system).

### 4.5. Statistical Analysis

To evaluate the main research question, analyses of variance were performed with period (anticipation, task, post-task) as the within-subjects factor, group (PE, UP, CO) as the between-subjects factor, and the respective cardiovascular variable as the dependent variable. Scores obtained at baseline were entered as covariates. That way, changes of cardiovascular variables produced by the stress manipulation were adjusted for baseline levels, ensuring that the analyzed residual scores were due to the acute stress, and not to individual differences in baseline levels. A further advantage of this approach, compared to simple change scores or the inclusion of baseline in the within-subjects factor, is that it controls for measurement error inherent in the use of repeated measures of the same kind [85,86,87]. If necessary, Greenhouse–Geisser corrections were used to adjust for nonsphericity of the variance–covariance matrices. In addition, the described statistical analyses were done within the group with a history of preeclampsia, with mild and severe as well as early, preterm, and term preeclampsia as the between-subjects factor. For graphical representation of the different time courses, standardized residualized change scores were calculated by linear regressions using the baseline period to predict the variables during the following periods, respectively [88,89]. These scores best represent those constituting the statistical results in the analyses of variance; i.e., they are adjusted for differences in baseline levels. One-way analyses of variance was performed to explore potential differences between the three groups in age, BMI, chronic stress, depression, and hemodynamic levels in resting conditions (at baseline). Further supplementary analyses were done to explore potential differences between groups in day of gestation, baby weight and height (independent *t*-tests), and parity (Chi-square test).

## Figures and Tables

**Table 1:** Hemodynamic variables (mean ± SD) of participants, and statistical results for group differences in response to the stress manipulation. CO: women without gestation during the last three years; UP: women with uncomplicated pregnancies; PE: women with a history of preeclampsia; BP: blood pressure; SVRI: systemic vascular resistance index.

	Baseline	Anticipation	Task	Post-Task	*F*-Statistics	
**Mean Arterial BP (mmHg)**						
CO	86.9 ± 9.4	89.7 ± 9.7	94.8 ± 10.5	90.3 ± 9.3	period	*F*(_1.7,207.2_) = 2.3, *p* = 0.108
UP	84.9 ± 8.4	87.0 ± 8.5	94.0 ± 9.4	89.5 ± 8.2	period x group	*F*(_3.5,207.2_) = 1.7, *p* = 0.149
PE	91.6 ± 10.0	92.7 ± 8.9	100.1 ± 10.7	95.5 ± 9.0		
**Systolic BP (mmHg)**						
CO	109.3 ± 11.0	112.8 ± 11.8	119.1 ± 12.7	113.3 ± 12.0	period	*F*(_1.2,200.7_) = 1.2, *p* = 0.307
UP	107.0 ± 9.6	109.6 ± 10.2	118.7 ± 12.0	112.2 ± 9.5	period x group	*F*(_3.5,200.7_) = 1.4, *p* = 0.243
PE	113.3 ± 11.8	115.2 ± 10.5	123.7 ± 12.6	118.2 ± 11.1		
**Diastolic BP (mmHg)**						
CO	70.9 ± 9.1	73.3 ± 8.8	77.5 ± 9.8	73.8 ± 8.5	period	*F*(_1.8,216.8_) = 3.5, *p* < 0.05
UP	69.0 ± 8.2	71.2 ± 7.9	76.8 ± 8.6	73.2 ± 7.6	period x group	*F*(_3.6,216.8_) = 1.9, *p* = 0.124
PE	75.4 ± 9.8	76.1 ± 8.9	82.6 ± 10.5	78.8 ± 8.3		
**Heart Rate (bpm)**						
CO	71.0 ± 10.9	73.6 ± 12.0	83.9 ± 15.0	72.1 ± 10.9	period	*F*(_1.3,153.2_) = 1.6, *p* = 0.208
UP	72.6 ± 7.6	74.2 ± 8.8	83.5 ± 10.5	75.5 ± 8.7	period x group	*F*(_2.6,153.2_) = 5.5, *p* < 0.01
PE	72.4 ± 9.3	74.5 ± 9.5	80.7 ± 11.7	74.9 ± 9.9		
**Stroke Index (mL/m^2^)**						
CO	43.0 ± 8.9	42.9 ± 8.6	42.4 ± 9.4	42.6 ± 8.2	period	*F*(_1.8,218.0_) = 1.7, *p* = 0.183
UP	41.6 ± 6.4	41.1 ± 6.2	40.0 ± 6.2	40.1 ± 6.5	period x group	*F*(_3.6,218.0_) = 0.8, *p* = 0.489
PE	38.2 ± 7.0	37.7 ± 7.0	37.3 ± 7.1	37.2 ± 6.9		
**SVRI (dyn·s·m^2^/cm^5^)**						
CO	2267 ± 541	2325 ± 548	2223 ± 589	2400 ± 561	period	*F*(_1.7,208.9_) = 2.3, *p* = 0.438
UP	2251 ± 514	2292 ± 506	2292 ± 524	2377 ± 534	period x group	*F*(_3.5,208.9_) = 1.7, *p* < 0.05
PE	2692 ± 650	2680 ± 614	2743 ± 660	2788 ± 625		

**Table 2:** Respiration rate and baroreflex sensitivity (mean ± SD) of participants, and statistical results for group differences in response to the stress manipulation. CO: women without gestation during the last three years; UP: women with uncomplicated pregnancies; PE: women with a history of preeclampsia.

	Baseline	Anticipation	Task	Post-Task	*F*-Statistics	
**Respiration Rate (breath/min)**						
CO	14.7 ± 3.8	14.9 ± 3.5	17.4 ± 3.3	15.4 ± 4.1	period	*F*(_1.7,205.9_) = 14.3, *p* < 0.001
UP	15.9 ± 3.2	16.4 ± 2.5	18.0 ± 2.8	16.0 ± 2.8	period x group	*F*(_3.4,205.9_) = 0.7, *p* = 0.565
PE	16.4 ± 4.2	16.4 ± 2.6	17.8 ± 2.4	16.2 ± 2.6		
**Baroreflex Sensitivity (ms/mmHg)**						
CO	14.4 ± 4.0	14.1 ± 3.8	11.9 ± 3.4	13.4 ± 3.8	period	*F*(_2,240_) = 1.7, *p* = 0.177
UP	13.4 ± 4.0	12.6 ± 3.1	11.8 ± 2.7	12.3 ± 3.2	period x group	*F*(_4,240_) = 2.1, *p* = 0.088
PE	14.0 ± 4.0	13.3 ± 3.4	11.7 ± 2.9	12.2 ± 4.2		

**Table 3:** Phase synchronization indices of the low-frequency (LF) components (mean ± SD) of participants, and statistical results for group differences in response to the stress manipulation. CO: women without gestation during the last three years; UP: women with uncomplicated pregnancies; PE: women with a history of preeclampsia; γ: synchronization index; SBP: systolic blood pressure, DBP: diastolic blood pressure; RRI: R–R intervals.

	Baseline	Anticipation	Task	Post-Task	*F*-Statistics	
**γ_SBPxRRI,LF_ (−)**						
CO	0.41 ± 0.21	0.48 ± 0.18	0.34 ± 0.13	0.41 ± 0.19	period	*F*(_2,240_) = 0.4, *p* = 0.675
UP	0.39 ± 0.17	0.41 ± 0.18	0.37 ± 0.15	0.42 ± 0.18	period x group	*F*(_4,240_) = 2.3, *p* = 0.058
PE	0.36 ± 0.17	0.39 ± 0.16	0.37 ± 0.16	0.38 ± 0.16		
**γ_DBPxRRI,LF_ (−)**						
CO	0.40 ± 0.19	0.45 ± 0.17	0.32 ± 0.13	0.40 ± 0.17	period	*F*(_2,240_) = 0.2, *p* = 0.821
UP	0.38 ± 0.15	0.38 ± 0.15	0.32 ± 0.13	0.40 ± 0.15	period x group	*F*(_4,240_) = 1.0, *p* = 0.428
PE	0.36 ± 0.15	0.40 ± 0.16	0.31 ± 0.16	0.36 ± 0.14		
**γ_SBPxDBP,LF_ (−)**						
CO	0.75 ± 0.13	0.79 ± 0.11	0.73 ± 0.13	0.75 ± 0.12	period	*F*(_1.9,226.3_) = 0.7, *p* = 0.481
UP	0.79 ± 0.12	0.78 ± 0.14	0.70 ± 0.17	0.78 ± 0.12	period x group	*F*(_3.8,226.3_) = 1.7, *p* = 0.219
PE	0.75 ± 0.13	0.79 ± 0.11	0.68 ± 0.15	0.74 ± 0.12		

**Table 4:** Phase synchronization indices of the high-frequency (HF) components (mean ± SD) of participants, and statistical results for group differences in response to the stress manipulation. CO: women without gestation during the last three years; UP: women with uncomplicated pregnancies; PE: women with a history of preeclampsia; γ: synchronization index; SBP: systolic blood pressure, DBP: diastolic blood pressure; RRI: R–R intervals; RESP: respiration.

	Baseline	Anticipation	Task	Post-Task	*F*-Statistics	
**γ_SBPxRRI,HF_ (−)**						
CO	0.60 ± 0.19	0.50 ± 0.20	0.33 ± 0.17	0.50 ± 0.25	period	F(_2,240_) = 0.6, *p* = 0.550
UP	0.63 ± 0.22	0.52 ± 0.21	0.33 ± 0.15	0.52 ± 0.22	period x group	F(_4,240_) = 1.0, *p* = 0.408
PE	0.66 ± 0.17	0.53 ± 0.19	0.29 ± 0.13	0.54 ± 0.20		
**γ_DBPxRRI,HF_ (−)**						
CO	0.40 ± 0.26	0.37 ± 0.20	0.29 ± 0.12	0.36 ± 0.23	period	F(_2,240_) = 8.4, *p* < 0.001
UP	0.36 ± 0.22	0.30 ± 0.20	0.29 ± 0.15	0.33 ± 0.17	period x group	F(_4,240_) = 1.3, *p* = 0.258
PE	0.37 ± 0.21	0.31 ± 0.21	0.22 ± 0.13	0.31 ± 0.20		
**γ_RESPxRRI,HF_ (−)**						
CO	0.71 ± 0.19	0.62 ± 0.18	0.39 ± 0.19	0.62 ± 0.22	period	F(_2,240_) = 0.5, *p* = 0.591
UP	0.69 ± 0.22	0.57 ± 0.20	0.33 ± 0.18	0.55 ± 0.26	period x group	F(_4,240_) = 0.5, *p* = 0.736
PE	0.70 ± 0.23	0.57 ± 0.25	0.32 ± 0.18	0.58 ± 0.22		
**γ_RESPxSBP,HF_ (−)**						
CO	0.67 ± 0.22	0.55 ± 0.20	0.30 ± 0.18	0.58 ± 0.24	period	F(_1.9,223.4_) = 1.1, *p* = 0.342
UP	0.70 ± 0.22	0.58 ± 0.19	0.27 ± 0.16	0.53 ± 0.26	period x group	F(_3.7,223.4_) = 2.2, *p* = 0.080
PE	0.68 ± 0.23	0.54 ± 0.24	0.22 ± 0.15	0.57 ± 0.25		
**γ_RESPxDBP,HF_ (-)**						
CO	0.33 ± 0.25	0.26 ± 0.18	0.18 ± 0.13	0.24 ± 0.19	period	F(_2,240_) = 3.0, *p* = 0.053
UP	0.33 ± 0.20	0.24 ± 0.15	0.17 ± 0.12	0.24 ± 0.16	period x group	F(_4,240_) = 2.2, *p* = 0.065
PE	0.28 ± 0.18	0.20 ± 0.19	0.11 ± 0.09	0.24 ± 0.19		

**Table 5:** Characteristics of the participants (mean ± SD, range), and statistical differences.

	PE (*n* = 35)	UP (*n* = 38)	CO (*n* = 51)	*p*-Value
Age (years)	33.7 ± 4.8, 25–42	32.4 ± 4.0, 26–44	32.4 ± 5.3, 25–44	*p* = 0.41
Height (cm)	168.2 ± 6.8, 153–182	167.5 ± 5.8, 157.5–179	168.4 ± 5.6, 156–180	*p* = 0.78
Weight (kg)	77.9 ± 15.7 **^3^**, 48.9–121.9	68.7 ± 11.5, 49.1–97.7	65.3 ± 10.5 **^1^**, 45–93	*p* < 0.001
BMI (kg/m^2^)	27.8 ± 5.9 **^2,3^**, 20.7–44.2	24.6 ± 4.6 **^1^**, 17–36.3	23.0 ± 3.2 **^1^**, 16.9–30.9	*p* < 0.001
Delivery (day)	253 ± 21, 197–287	278 ± 10, 254–291	–	*p* < 0.001
Baby height (cm)	46.9 ± 5.1, 31–57	51.3 ± 1.8, 47–56	–	*p* < 0.001
Baby weight (g)	2568 ± 853, 800–3940	3404 ± 341, 2780–4010	–	*p* < 0.001

**^1,2,3^** Denotes a significant difference between PE (^1^), UP (^2^), and CO (^3^) based on Bonferroni-corrected post-hoc tests. BMI: body mass idex.

## Abbreviations

BP	Blood pressure
BRS	Baroreflex reflex sensitivity
CO	Women without gestation during the last three years
DBP	Diastolic blood pressure
MAP	Mean arterial pressure
PE	Women with a history of preeclampsia
SBP	Systolic blood pressure
SI	Stroke index
SVRI	Systemic vascular resistance index
UP	Women with uncomplicated pregnancies
